# Age-based stereotype threat effects on dynamic balance in healthy older adults

**DOI:** 10.3389/fnsys.2024.1309158

**Published:** 2024-08-07

**Authors:** Liliane Borel, Béatrice Alescio-Lautier, Jacques Léonard, Isabelle Régner

**Affiliations:** Aix Marseille Univ, CNRS, CRPN, Marseille, France

**Keywords:** stereotype threat, reduced stereotype, older adults, postural control, task difficulty, increased attentional load

## Abstract

**Introduction:**

Stereotype threat can lead older adults to perceive their experiences in a biased manner, giving rise to interfering thoughts and negative emotions that generate stress and anxiety. Negative beliefs about aging may serve as an additional factor that increases the need for attentional demand, potentially resulting in a performance level below their actual capabilities. In the present study, we asked whether negative aging stereotypes influence a dynamic balance task and explored the means to counteract them in healthy elderly participants.

**Methods:**

The performance of balance was compared in two groups of participants aged 65 to 75 years (*n* = 22) under stereotype threat or reduced-threat situation. Balance abilities were tested under dynamic conditions, requiring participants to maintain balance on a moving platform and using a gradient of difficulty (with eyes open or closed, without or with foam). Postural performance was evaluated by means of posturographic evaluation of the center of pressure displacement and motion analysis. Additionally, we investigated the effects of stereotype threat on a preferred walking speed task and on the Timed Up and Go (TUG) test.

**Results:**

Participants under stereotype threat showed poorer balance, particularly in challenging conditions (eyes closed, on foam), with less effective body segments stabilization. Their postural stabilization on foam was worse compared to a solid surface. Conversely, those in the reduced threat condition maintained better body segment stabilization across all conditions, indicating consistent postural control regardless of the presence of foam. Stereotype threat did not affect preferred walking speed or the time to complete the “Time Up and Go” test.

**Discussion-conclusion:**

This study provides the first description of age-based stereotype threat effects on a dynamic balance task and how to counteract them in healthy older adults. We suggest that the decrease in postural performance observed in participants exposed to stereotype threat can be attributed to a split in attentional focus between negative intrusive thoughts and the attention needed for maintaining balance. These findings open new perspectives on how to overcome negative expectations when evaluating and training physical abilities, thereby contributing to fall prevention among older adults.

## Introduction

Balance and posture control are typically basic coordinated actions based on the integration of multiple sensory systems, and largely automatic (Massion, 1994; Kolb et al., 2001). However, under challenging conditions, when tasks become complex, balance performance may be altered. In older individuals, many physiological changes of the different sensory systems (visual, vestibular, proprioceptive, and haptic) along with changes in the motor system (reduced muscle strength and changes in the central motor commands) can alter balance abilities (for a review, see Sturnieks et al., 2008; Borel and Alescio-Lautier, 2014). In challenging conditions, older adults have more difficulty maintaining their balance, which can represent a threat to their posture and lead to fear of falling (Almajid et al., 2020). Postural threat can be induced both by external and internal changes. Namely, by reducing the sensory information available to maintain balance (excluding vision or disrupting proprioceptive and vestibular information) and placing the subject in dynamic conditions. Another way is to modify the internal representation of the postural task. This can be achieved by placing the subjects on an elevated platform. In such situation, the fear of falling can disturb postural performance (Carpenter et al., 2006; Davis et al., 2009; Young et al., 2012). These various situations lead to a shift in maintaining balance from automatic control to cognitive supervision. The theoretical notion of supervisory control involves attentional processes that become increasingly important depending on the complexity of the postural task and the context in which it occurs. An increased attentional demand has been reported even under relatively simple conditions (for a review, see Woollacott and Shumway-Cook, 2002). Increased attentional demand has also been observed due to environmental situations where visual, vestibular, or proprioceptive sensory information is perturbed (Teasdale and Simoneau, 2001). Changes in attention focus with attempts to consciously control posture have been reported in threatening postural condition (e.g., unpredictable support surface translation during stance, Johnson et al., 2019).

The present study is based on the idea that an increase in attentional load may also be due to age-based stereotype threat (ABST), a phenomenon where negative beliefs about aging impact older adults’ cognitive performance (Lamont et al., 2015; Swift et al., 2021). ABST is rooted in negative stereotypes that aging inevitably leads to severe decline in abilities. These stereotypes are likely to heighten interfering thoughts, anxiety and stress, leading to underperformance in task domain associated with these beliefs (Steele and Aronson, 1995; Steele, 1997; Schmader et al., 2008).

The impact of social stereotypes has been particularly studied on the cognitive performance of older adults. Several studies have shown that ABST can significantly impair memory performance in healthy older adults, demonstrating that the conditions under which memory tests are conducted can lead them to perform below their true abilities (for reviews see Lamont et al., 2015; Armstrong et al., 2017). For example, simply emphasizing the memory component of a test increased performance differences between age groups, while de-emphasizing it or presenting the test as age-fair eliminated these differences (Rahhal et al., 2001; Chasteen et al., 2005; Desrichard and Köpetz, 2005). Likewise, Mazerolle et al. (2012, 2015) have shown that mentioning the presence of younger adults in the study led to both impaired working memory capacity and increased inappropriate cognitive automatisms among healthy older adults. It is thus now well-established that ABST can contribute to age-related differences in memory tasks (for recent reviews, see Barber, 2020; Barber and Liu, 2020).

Regarding posture and gait control, the impact of ABST is still relatively underexplored. Few studies have examined the influence of aging stereotypes on walking speed and functional performances such as chair lifting (e.g., Hausdorff et al., 1999; Levy and Leifheit-Limson, 2009; Levy et al., 2014). The authors showed that the reinforcement of negative stereotype of aging (e.g., ‘senile’, ‘dependent, and ‘diseased’) did not change gait parameters whereas the subliminal implicit activation of positive aging stereotypes (e.g., ‘wise’, ‘astute’, and ‘accomplished’) significantly improved older adult performances. Similarly, Marquet et al. (2018) reported no effects of ABST on various physical performance measures, such as walking and the Timed Up and Go (TUG) test. An ABST effect was only observed for tandem walking, which involves walking heel-to-toe. The impact of stereotype threat on older adults’ walking performance was found to depend on the task’s difficulty, such as walking on a narrow base of support (Barber et al., 2020). These authors suggested that stereotype threat consumes cognitive resources, which in turn leads to performance decrements.

To the best of our knowledge, to date, no study has examined the potential effects of stereotype threat on dynamic balance abilities on a moving platform. Only Chiviacowsky et al. (2018) have emphasized the role of aging stereotypes on balance learning in older adults. They reported reduced performance in learning new balance task in older adults receiving a negative age stereotype. Considering the effects of ABST on balance performance could become crucial when assessing actual balance capabilities in older adults, particularly in diagnostic or rehabilitation monitoring contexts.

Testing ABST effects requires comparing older adults’ performance in at least two testing conditions (Steele and Davies, 2003; Spencer et al., 2016; Mazerolle et al., 2020): a Threat condition, where very subtle cues are sufficient to activate negative aging stereotypes (e.g., emphasizing the memory component of a test, noting the presence of younger participants in the study without mentioning age differences), and a Reduced-Threat condition, where active measures must be taken to minimize the stereotype’s influence (e.g., age-fair instructions, providing positive information about aging and memory preservation). It is important to note that what is usually considered as a control condition in experimental design (i.e., a condition without any intervention of the researcher), cannot be a reduced-Threat condition. Simply presenting a memory test as a memory test, does not constitute a reduced-ABST condition but rather a subtle activation of ABST (Mazerolle et al., 2020). Action is required to ensure eliminating that subtle cues in the testing environment activate aging stereotypes.

The present study aimed to investigate the potential impact of negative aging stereotypes on balance abilities and to explore the effect of an intervention designed to alleviate this threat in healthy older adults. Specifically, balance abilities were tested under dynamic conditions, requiring participants to maintain balance on a moving platform. In this context, we addressed the question whether ABST effects depend on task difficulty. To achieve this, we reduced the available sensory information by: (1) instructing participants to close their eyes and (2) placing them on a foam pad. Maintaining a stable posture on an unstable surface with closed eyes becomes particularly challenging since these conditions alter the proprioceptive and haptic feedback using foam. This is reinforced by the fact that older adults have an increased dependency on visual information to maintain balance (e.g., Borger et al., 1999; Ruffieux et al., 2015; Reimann et al., 2020). We hypothesized that balance performance would decline when healthy older adults are exposed to stereotype threat compared to a reduced threat situation, and that this effect would intensify as the difficulty of the condition increases. Balance performance was analyzed using global postural stabilization and body segment stabilization. In previous studies, the stabilization of upper body segments, particularly the head, has been found to be more sensitive to changes in the external or internal states of participants than global postural stabilization (Tardieu et al., 2009; Young et al., 2012; Borel and Ribot-Ciscar, 2016). Therefore, in the present study, we propose that the influence of negative aging stereotypes on balance performance may affect the stabilization of body segments more significantly than global postural stabilization.

To compare our data with previous studies from the literature, participants were asked to perform a preferred walking speed task. They were also required to perform a Time Up and Go test (TUG) which is an indicator of balance performance (Podsiadlo and Richardson, 1991). Under these experimental conditions, we expect that stereotype threat would have minimal or no effect on our healthy older adult participants.

To test our hypotheses, participants were pseudo-randomly assigned into two groups. In the stereotype threat group participants were told that the experiment was aimed at evaluating how young and older adults maintain their balance. The same information was given to the reduced stereotype group, in which participants were also told that performances on these tasks usually do not differ between younger and older adults (i.e., age-fair instructions). The relevance of such age-fair instructions has been demonstrated in the context of stereotype threat on the memory performance of older adults (e.g., Mazerolle et al., 2012, 2015).

## Method

### Participants

Twenty-two healthy participants (see Statistical analysis section for *a priori* sample size calculation) aged 65 to 75 years were pseudo-randomly assigned to the Stereotype Threat (ST) group (11 participants aged 70.0 ± 3.6 (mean ± SD); 9 females and 2 males) versus the Reduced Threat (RT) group (11 participants aged 70.6 ± 3.5; 10 females and 1 male). All the participants were included based on the following criteria: no previous physical, neurological, sensory, or cognitive disorders, no postural and gait disorders, no history of unprovoked falls in the previous 12 months, and absence of medication that might influence their balance or cognitive performance. All the participants were right-handed and were either emmetrope or wore corrective glasses. Each gave informed consent to this study, which was approved by the local ethics committee.

### Procedure

The participants were required to respond to questions regarding frailty criteria adapted from Fried et al. (2001) and a Mini-Mental State Examination (MMSE) (Folstein et al., 1975) to assess their gait and cognitive status, respectively. Any positive response among the 5 frailty criteria, or a score lower than 26 out of 30 on the MMSE, were criteria for non-inclusion. The participants were pseudo-randomly assigned to either Stereotype Threat (ST) or Reduced stereotype Threat (RT) group. The two groups of participants did not differ in terms of their gait and cognitive status (Table 1). The participants were equipped with active markers for motion analysis evaluation. In the ST condition, participants were told that the experiment was aimed at evaluating how young and older adults maintain their balance. In the RT condition, the same information was given, but each participant was also told that performance on these tasks usually does not differ between younger adults and older adults. These instructions were given three times to sustain their effect throughout the experiment. Postural performance was examined in dynamic condition under different levels of difficulty (and without vision, with and without foam). Finally, participants were assessed while performing a six-meter preferred walking speed task and the Time Up and Go test.

**Table 1:** Description of the two groups.

	Threat condition		Reduced-threat condition	
	*M*	SD	*M*	SD
MMSE	28.73	1.35	28.73	1.19
Average velocity (m/s)	1.22	0.13	1.21	0.16
TUG moyen (s)	8.38	1.35	9.1	1.04
Age (years)	70	3.61	70.55	3.45
Span	3.27	0.06	3.09	0.32

## Material and measures

### Postural task

The participants received instructions to stand quietly, barefoot, assuming a natural stance with their feet placed shoulder-width apart on a force plate mounted on a translator (Synapsys, Marseille, France). All postural tests were performed under dynamic conditions. The participants were asked to keep their balance on the platform which moved sinusoidally fore and aft at 0.5 Hz with an amplitude of 7 cm. Twelve oscillation cycles were completed per trial. A safety railing encircled the platform to prevent participants from falling in case they lost their balance. Recordings of 25.6 s duration were performed under four crossed conditions: two visual conditions (eyes open: EO; eyes closed: EC) and two postural conditions (with foam; without foam). During condition with foam, balance performance was evaluated as participants stood on a 6-cm-thick foam pad (Airex Balance Pad); this condition was more challenging than the condition without foam and generated larger body oscillations. The condition without foam always preceded the condition with foam to avoid anxiety related to maintaining balance on the foam and to prevent muscular fatigue associated with this more demanding postural condition. Within each postural condition, the two visual conditions (EO, EC) were run in a random order. The different experimental conditions (without foam-EO, without foam-EC) and (with foam-EO, with foam-EC) were repeated three times. To rule out the effect of fatigue, a 30-s delay was incorporated between each successive trial. During this interval, participants were instructed to move their ankles and knees. Furthermore, after every four trials, a 5-min rest period was provided, during which the participants sat on a chair.

### Preferred walking speed task and time up and go test

The study also assessed the impact of instructions related to stereotype threat or reduced threat on basic mobility and functional performance. This evaluation included the assessment of walking speed and the Time Up and Go (TUG) test. Walking speed was measured at the participant’s preferred velocity over a distance of six meters. The time taken to complete the entire sequence of the TUG test was recorded. The test started with the participant in a seated position on a chair, then they were instructed to stand up without using their arms for support, walk a distance of 3 meters, turn around, and return to the seated position. Both the preferred walking speed task and the TUG test were repeated three times.

### Data acquisition and processing

Postural control was assessed by means of posturography and motion analysis evaluations.

#### Posturographic evaluation of postural performance

**Figure 1:**
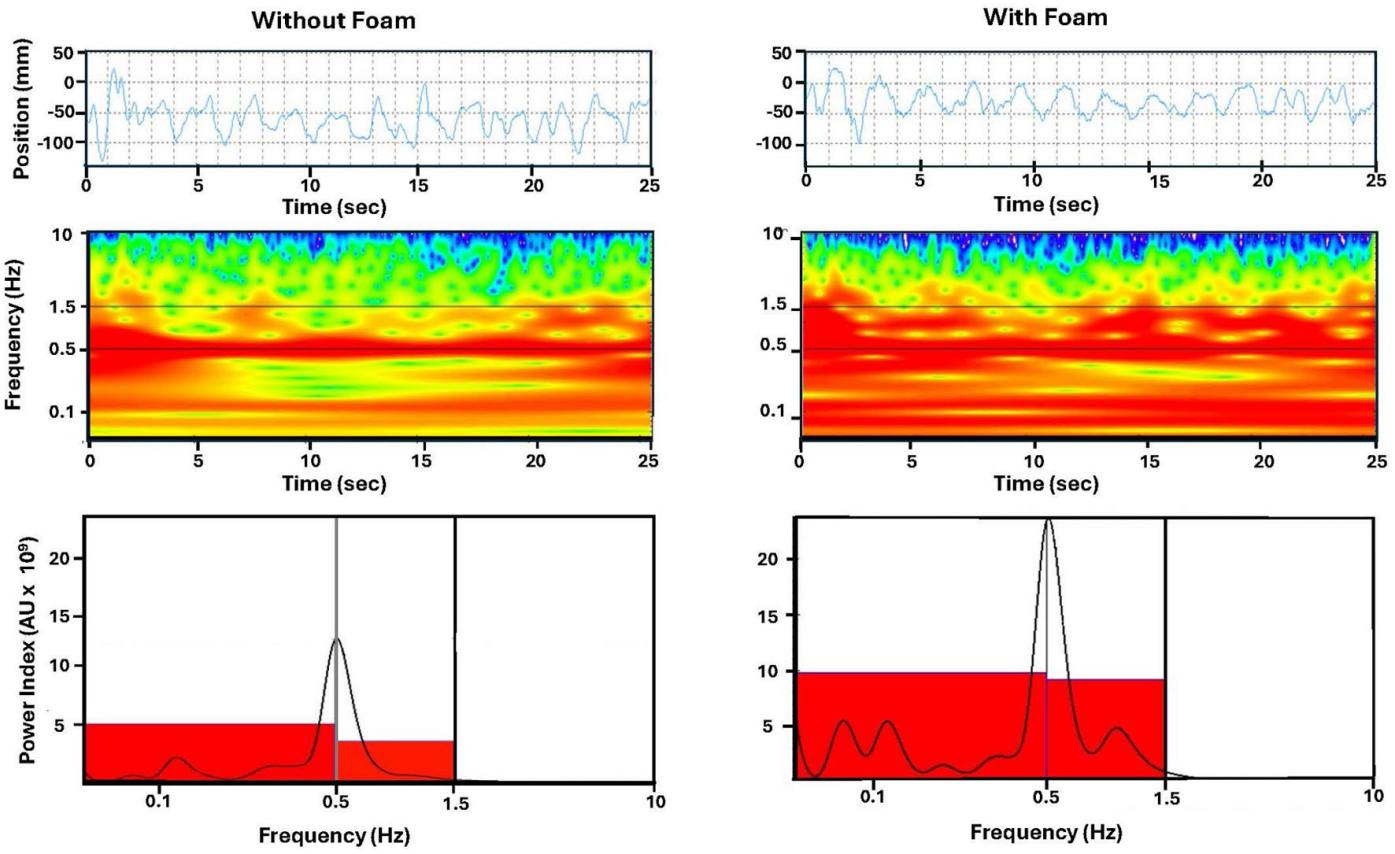
Posturographic recording analysis. Top: displacement of the center of pressure in the anteroposterior direction. Middle: three dimensional chart obtained by wavelet analysis with time represented in abscissa, frequency on the ordinate. Spectral power density is color coded. Bottom: spectral power density versus frequency plot. The red rectangles illustrate the mean values of the spectral power density in each frequency band. Example for a participant tested in stereotype threat condition, eyes closed, on foam (right part) an without foam (left part).

Postural stabilization was assessed by quantifying the displacement of the Center of Pressure (CoP) in the antero-posterior direction. The CoP displacement was sampled at 100 Hz for 25.6 s. The recordings were processed using a wavelet transformation which is a nonlinear analysis of the CoP displacement processing of the posturography data (Lacour et al., 2008; Young et al., 2012; Borel and Ribot-Ciscar, 2016). The wavelet software (PosturoPro, Framiral, Cannes, France) allows the visualization of the change in the frequency components of body sway with time (Figure 1). The wavelet transformation method provides a three-dimensional representation of body sway: the CoP displacement frequency as a function of time, and as a third dimension, the spectral power is represented by a color code. Postural performance was evaluated through the spectral power density of the recorded signal within three frequency bandwidths (0.05–0.5 Hz, 0.5–1.5 Hz, and 1.5–10 Hz), arbitrary unit: AU, which correspond to the slowest, medium, and highest movements, respectively. These measures reflected the energy expended for postural stabilization. A higher spectral power density value indicates more significant participant movements and heightened instability.

#### Motion analysis evaluation of postural performance

Body segment stabilization was analyzed using motion recordings; it was measured simultaneously with the postural recordings of CoP displacements. Body segment stabilization was recorded using a video motion analyzer (Codamotion, Charnwood Dynamics, UK) sampled at 100 Hz. Five active markers were placed on the head, shoulder, hip, knee, and ankle on one side of the body. Body segment stabilization was defined as the standard deviation (mm) of each active marker and calculated in the antero-posterior plane using a locally developed program. An increased standard deviation indicates larger displacements of the body segments and decreased stability of the body segments in space.

### Statistical analysis

As our main hypothesis focused on postural performance as a function of stereotype threat (between-subject factor) and postural task complexity (within-subject factor), we determined the necessary sample size for conducting mixed ANOVAs to analyze the standard deviation of body segments (head, shoulder, hip, knee, ankle) and spectral power density at three different frequencies bandwidths (first, second, and third). Given that the total sample size typically decreases as the number of measurements increases, we opted for a cautious approach and calculated *a priori* sample size based on the three measurements of spectral power density, rather than the five measurements of standard deviation. In a relevant meta-analysis conducted by Lamont et al. (2015) on age-based stereotype threat, they reported an effect size of *d* = 0.52 when using stereotype-based instructions, like those employed in our studies. Using this reported effect size (equivalent to *f* = 0.25), a significance level of 0.05, a power level of 0.80, considering two groups (the between-subject factor), and accounting for 6 repeated measurements (3 levels of frequency bandwidths *2 levels of postural condition: with or without foam), the *a priori* power analysis indicated that a total sample size of 20 participants would be adequate to detect the critical interaction between group and the repeated measures associated with the postural task. To anticipate attrition rate, we tried to include more participants but were only able to recruit 22 older adults. One participant had missing postural data, leading to a final sample of 21 older adults.

Parameters describing postural performance (standard deviation and spectral power density), were analyzed using planned contrast analyses for mixed design. They were performed separately for EO and EC conditions, using the group (ST group versus RT group) as the between-subject factor, and postural condition (with foam versus without foam) and body segments (head, shoulder, hip, knee, ankle) for standard deviation or frequency bandwidths (first, second, and third) for spectral power density as the within-subject factors. All the results were considered statistically significant at *p* < 0.05.

## Results

### Body segment stabilization

**Figure 2:**
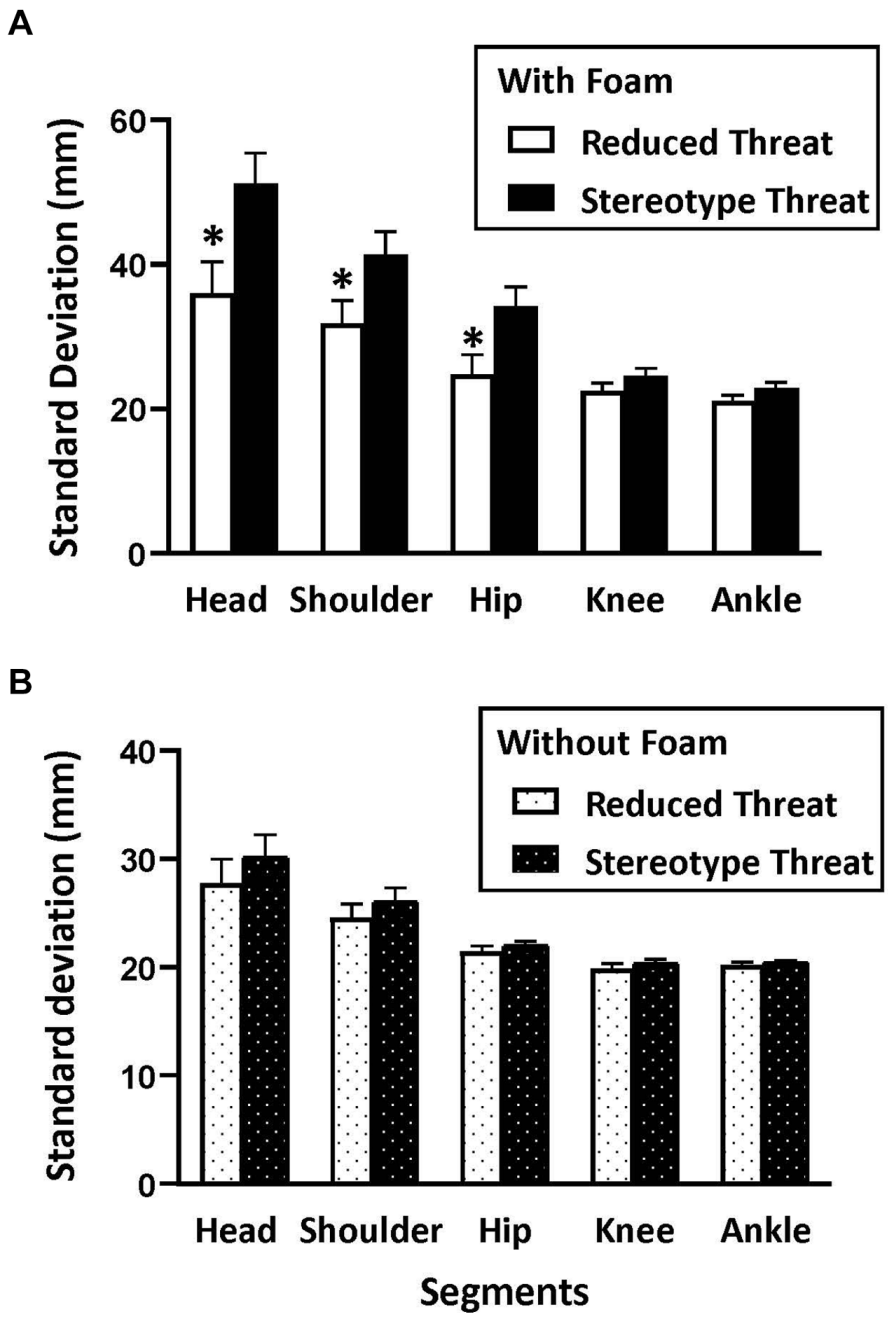
Influence of age-related stereotype on body segments stabilization in the eyes closed condition. Body segments standard deviations compared for Stereotype Threat (ST) and Reduced Threat (RT) groups, with foam (panel **A**) and without foam (panel **B**). Error bars represent the Standard Error of the Mean (SEM). *indicates statistical significance between ST and RT groups (*p* < 0.05).

The mixed ANOVA performed under the EC condition on the standard deviation of body segments first indicated a main effect of group [*F*(1,19) = 5.69, *p* < 0.03, *η^2^*_p_ = 0.23], of foam [*F*(1,19) = 40.10, *p* < 0.001, *η^2^*_p_ = 0.68], and of body segments [*F*(4,76) = 42.98, *p* < 0.001, *η^2^*_p_ = 0.69]. The main effect of the body segments was mainly driven by a linear trend [*F*(1, 19) = 52.71, *p* < 0.001, *η^2^*_p_ = 0.75]: There was a gradual increase in body segment movement from the ankle to the head, indicating better spatial stabilization in the lower body segments. More importantly and as expected, *a priori* contrast analysis indicated significant differences in the stabilization of body segments between the two groups only when participants were on foam (Figure 2). Specifically, in the condition with foam, participants in the ST group showed poorer stabilization compared to those in the RT group both for the head [*F*(1,19) = 6.49, *p* = 0.020, *η^2^*_p_ = 0.26], shoulders [*F*(1,19) = 4.56, *p* = 0.046, *η^2^*_p_ = 0.19], and hips [*F*(1,19) = 6.17, *p* = 0.022, *η^2^*_p_ = 0.25]. It is noteworthy that the corresponding omnibus postural condition x body segments x group interaction was significant [*F*(4,76) = 5.03, *p* < 0.001, *η^2^*_p_ = 0.21].

Also as expected, the similar mixed ANOVA performed under the EO condition did not produce any significant results.

### Postural stabilization

The mixed ANOVA conducted under EC condition on the wavelet transformation data first revealed a main effect of the mean spectral power density [*F*(2,38) = 20.40, *p* < 0.001, *η^2^*_p_ = 0.52], and a main effect of foam [*F*(1,19) = 14.05, *p* = 0.001, *η^2^*_p_ = 0.43]. The main effect of foam showed a higher spectral power density during tests performed with foam compared to those without foam. This supports an increased energy cost for anteroposterior stabilization with foam, indicating more challenging postural control. Contrary to the findings on standard deviation, the main effect of group did not reach significance here.

**Figure 3:**
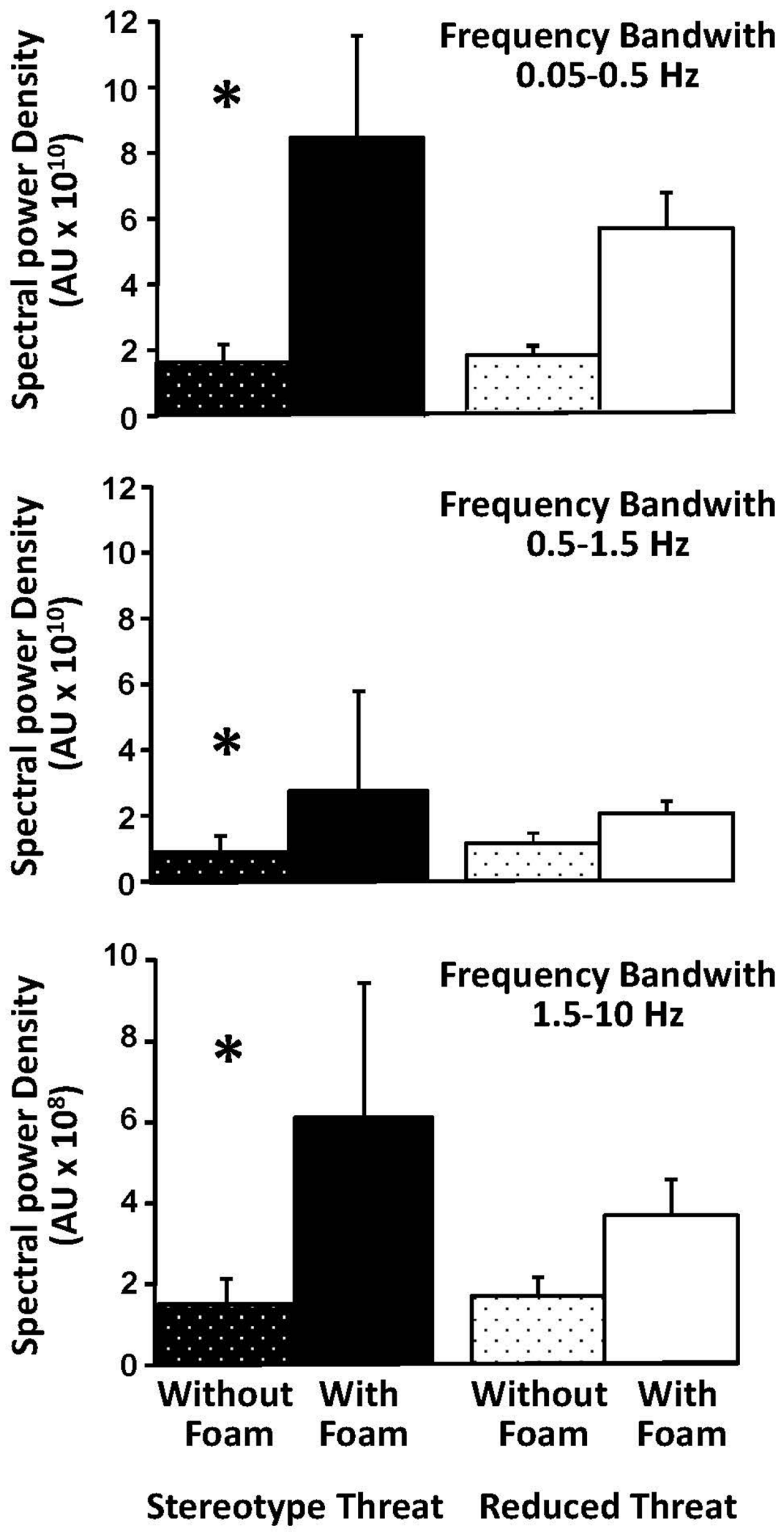
Influence of age-related stereotype on postural stabilization in the eyes closed condition. Spectral power density in the three frequency bands compared for each postural condition (with foam and without foam) in the Stereotype Threat group (left part) and the Reduced Threat group (right part). Error bars represent the Standard Error of the Mean (SEM). * (*p* < 0.05).

However, as expected and similarly to what was obtained on standard deviation, planned contrast analyses supported the influence of stereotype threat: higher spectral power density was observed with foam relative to without foam (Figure 1) in the ST group for all three frequency bands [F(1,19) = 13.37, *p* = 0.002, *η^2^*_p_ = 0.41], [F(1,19) = 10.45, *p* = 0.004, *η^2^*_p_ = 0.36] and [F(1,19) = 6.35, p = 0.02, *η^2^_p_* = 0.25] (Figure 3), whereas no such differences reached significance in the RT group. These findings indicate that participants in the ST group exhibited decreased body segment stabilization compared to those in the RT group when task difficulty increases. Unlike the contrasts on standard deviations that were sustained by the corresponding significant interaction, the interaction for spectral power density was not significant.

As previously, the mixed ANOVA did not yield any significant results only in the EO condition.

### Preferred walking speed task and time up and go test

Analysis using an independent samples *t*-test revealed no effect of stereotype threat on the participants’ performance regarding both the Time Up and Go (*M* = 8.3 s; SE = 1.4 s in the ST group and *M* = 9.1 s; SE = 1.0 s in the RT group) and the participants’ preferred walking speed (*M* = 1.22 m/s; SE = 0.13 m/s in the ST group and *M* = 1.21 m/s; SE = 0.16 m/s in the RT group).

## Discussion

This study explored the impact of age-based stereotype threat (ABST) on maintaining balance on a moving platform and examine an intervention aimed at reducing the threat effect in healthy older adults. Participants’ performance was evaluated by increasing the difficulty of the balance task through two visual conditions (eyes open and eyes closed) and two postural conditions (standing on a solid surface and on foam). The hypothesis was that balance performance would deteriorate when healthy older adults were exposed to ABST, and that this effect would intensify with increasing task difficulty. Additionally, we investigated the effects of ABST on walking task at preferred speed and on the Timed Up and Go (TUG) test. Under these experimental conditions, the hypothesis was that ABST effects would be minimal or even nonexistent.

Participants exposed to stereotype threat conditions exhibited poorer balance performance compared to those in the reduced-threat condition. When required to maintain a stable posture on foam, participants exposed to stereotype threat exhibited less effective body segments stabilization, as evidenced by greater displacements of body segments (specifically, the head, shoulders, and hips), compared to participants in the reduced threat condition. Among participants exposed to stereotype threat conditions a poorer postural stabilization was obtained when maintaining balance on foam compared to a solid surface. This was evidenced by an increase in spectral power density within three frequency bandwidths, indicating a higher energy cost required for stabilization. Following research exploring moderators and mediators of stereotype threat effects (Schmader et al., 2008; Lamont et al., 2015; Pennington et al., 2016), it is possible that the stereotype threat condition (i.e., mentioning the presence of both younger and older adults) induced changes in the demands placed on posture control. This may have led to intrusive thoughts and heightened anxiety, ultimately distorting the participants’ perception of the postural task.

Furthermore, in line with our hypothesis, the detrimental effect of stereotype threat becomes evident under the most challenging postural condition, specifically when older adults had their eyes closed and stood on foam. Regarding posture and balance, multisensory integration models highlight the importance of the simultaneous and congruent use of sensory inputs (visual, vestibular, proprioceptive) and their contribution to forming an internal model. These models are based on the concept of weighting these sensory inputs, which varies depending on the age of the population and the environmental context (e.g., Ernst and Banks, 2002; Kording et al., 2007; Dokka et al., 2010; Young et al., 2012). Specifically, older adults rely more heavily on visual information to maintain balance (Borger et al., 1999; Reimann et al., 2020). In challenging balance situations, when task demands increase, the concept of supervisory control becomes relevant, engaging attentional processes. Moreover, in older adults, attentional resources contribute more significantly to maintaining balance (for a review, see Woollacott and Shumway-Cook, 2002; Boisgontier et al., 2013; Borel and Alescio-Lautier, 2014). Therefore, in our experimental conditions, where visual, proprioceptive, and haptic cues essential for postural balance are altered (e.g., maintaining balance with eyes closed or standing on foam), it can be inferred that stereotype threat is consuming a portion of the attentional resources needed to maintain balance and distort the participant perception of postural task. Moreover, negative intrusive thoughts may lead to a split in attentional focus between these thoughts and the attention needed for maintaining balance. This could account for the decrease in postural performance observed when compared to the reduced threat group. These findings align with previous studies showing that stereotype threat has a greater impact on physical outcomes in older adults when the task becomes more challenging. This has been demonstrated in demanding gait tasks, such as walking within a narrow base of support (Barber et al., 2020) or performing a tandem walking test that necessitates walking heel-to-toe (Marquet et al., 2018). These authors suggest that older adults who are exposed to negative information about aging are more likely to adopt a cautious strategy when performing physical tasks, potentially disrupting their performance. Changes in the demands placed on posture control, with attentional resources primarily directed toward postural activity, have also been documented in postural threat conditions induced by elevating a moving platform off the floor (Carpenter et al., 2006; Young et al., 2012) or by receiving an unpredictable perturbation to balance (Johnson et al., 2019). Consequently, the redirection of attentional resources toward postural control appears to be a common change in response to threatening conditions, regardless of their origin.

In contrast, the reduced threat group showed better stabilization of body segments compared to the threat group, and postural stabilization remained consistent regardless of the presence of foam. These findings emphasize the presence of notably efficient postural control within this group. These results suggest that the age-fair instructions used to reduce stereotype threat offered a release from such pressure, allowing a more accurate evaluation of older adults’ actual performance. Therefore, it appears that the instructions provided to these participants were robust enough to overcome negative expectations related to our challenging postural conditions. Our results confirm the relevance of age-fair instructions in the domain of postural balance, as primarily demonstrated in the context of stereotype threat on the memory performance of older adults (e.g., Mazerolle et al., 2012, 2015). These authors reported the elimination of performance differences between young and older individuals in memory tasks. Interestingly, research studies that have primarily investigated the effects of positive stereotypes converge on the concept of age-related stereotypes influencing the physical performance of older adults (Hausdorff et al., 1999; Levy et al., 2014). These authors have demonstrated that the reinforcement of positive stereotypes of aging through subliminal priming significantly increased walking speed and the time to rise from a chair in older adults. They strongly support the idea that aging stereotypes influence physical performance.

The absence of stereotype threat effects in less challenging postural conditions (such as maintaining balance on a moving platform with eyes open, with and without foam) confirms the major role of visual cues in maintaining balance. As the task becomes easier, we can assume that it requires less attention and that the attentional demands involved in performance decrease. For our healthy older participants, the control level of these tasks might be relatively low, with much of the regulation being automated. Similarly, we found no impact of stereotype threat on participants’ preferred walking speed or on their performance in the Time Up and Go test. This may be attributed to the task demands not being sufficiently challenging. Our results align with previous studies investigating the negative effects of stereotype threat on gait performance in older adults, as they have consistently shown no significant effects of the stereotype threat manipulation on preferred walking speed (Hausdorff et al., 1999; Marquet et al., 2018; Barber et al., 2020).

### Limitations

The first limitation concerns the small sample size. Although our sample size was determined based on an *a priori* analysis and our findings are significant, the impact of the present study is limited by the relatively small sample size. The second limitation concerns the intervention used to activate ABST. We followed previous ABST studies in cognitive domains, using a subtle activation of negative aging stereotypes by noting the presence of younger adults without mentioning age differences. It is worth considering whether ABST effects would still be observed with another subtle intervention, such as simply mentioning the domain being evaluated (here “dynamic balance ability”). Given that ABST effects have been found in memory studies when tests are simply presented as memory tests, we are confident that presenting the test as a balance test should similarly induce stereotype threat. However, this hypothesis needs to be tested. Regardless of the method used in the threat condition, it is important to note that the reduced-threat condition requires active external intervention to neutralize the influence of negative aging stereotypes.

## Conclusion

In conclusion, these new findings shed light on the influence of negative aging stereotypes on balance performance in healthy older adults. They also emphasize the importance of addressing and mitigating these stereotype threat effects. Specifically, reducing the evaluative pressure during postural assessments can help reveal older adults’ true abilities. We suggest that stereotype threat situations may provide a theoretical framework for understanding some of the falls experienced by older adults, particularly those with a history of falls. Our results open new perspectives for addressing negative expectations when evaluating and training physical abilities, thereby aiding in fall prevention among older adults. Further investigation will examine the impact of negative aging stereotypes on balance performance in common daily-life situations where attentional resources are divided (e.g., cognitive-postural dual-task situations).

## Data availability statement

The original contributions presented in the study are included in the article/supplementary materials, further inquiries can be directed to the corresponding author.

## Ethics statement

The studies involving humans were approved by Comité de Protection des Personnes Sud Méditerranée I. The studies were conducted in accordance with the local legislation and institutional requirements. The participants provided their written informed consent to participate in this study.

## Author contributions

LB: Conceptualization, Formal analysis, Funding acquisition, Investigation, Methodology, Supervision, Writing – original draft. BA-L: Conceptualization, Funding acquisition, Methodology, Validation, Writing – review & editing. JL: Conceptualization, Investigation, Methodology, Writing – review & editing. IR: Conceptualization, Formal analysis, Funding acquisition, Methodology, Supervision, Writing – review & editing.
